# The Sphenoparietal Sinus Revisited: Anatomical and Histological Study With Application to Interventional Procedures and Skull Base Surgery

**DOI:** 10.7759/cureus.33419

**Published:** 2023-01-05

**Authors:** Grace Posey, Viktoriya Grayson, Juan J Cardona, Joshua Hanna, Marko Konschake, Arthur Wang, Grzegorz Wysiadecki, Joe Iwanaga, Aaron S Dumont, R. Shane Tubbs

**Affiliations:** 1 Department of Medicine, Tulane University School of Medicine, New Orleans, USA; 2 Department of Neurosurgery, Tulane University School of Medicine, New Orleans, USA; 3 Department of Anatomy, Histology and Embryology, Institute of Clinical and Functional Anatomy, Medical University of Innsbruck (MUI), Innsbruck, AUT; 4 Department of Normal and Clinical Anatomy, Medical University of Lodz, Lodz, POL; 5 Department of Neurology, Tulane Center for Clinical Neurosciences, Tulane University School of Medicine, New Orleans, USA; 6 Department of Structural and Cellular Biology, Tulane University School of Medicine, New Orleans, USA; 7 Department of Anatomical Sciences, St. George’s University, St. George’s, GRD; 8 Department of Surgery, Tulane University School of Medicine, New Orleans, USA; 9 Department of Neurosurgery, Ochsner Neuroscience Institute, Ochsner Health System, New Orleans, USA

**Keywords:** cadaveric study, anatomy, dural venous sinus, neurosurgery, skull base, sphenoparietal sinus

## Abstract

Background

The sphenoparietal sinus (SPS) is implicated in various clinical pathologies, specifically arteriovenous fistulas and venous sinus thrombosis. This study is aimed to better understand this venous structure of the skull base via histological examination.

Methods

Ten embalmed and latex-injected adult body donors' heads (20 sides) underwent microdissection of the SPS using a surgical microscope. The entire dura on the underside of the lesser wing of the sphenoid bone encompassing the region known as the groove for the SPS was harvested from each body donor and submitted for histological analysis (H&E, Periodic acid-Schiff [PAS], Masson's Trichrome). Five left and five right transverse sinuses were harvested and analyzed histologically as controls.

Results

A definitive SPS was identified in 14/20 (70%) of the latex-injected body donors. When present, the sinuses were classified as small, medium, or large. Tributaries included the middle meningeal veins, superficial Sylvian vein, and anterior temporal veins. All sinuses drained medially into the cavernous sinus. For the body donors analyzed histologically, 17 (85%) were consistent with a dural venous sinus and not a vein and were observed to have a rich nerve and arterial supply within their walls. The histological findings of the SPS were similar to those seen for the transverse sinus. The combined prevalence for the SPS in gross and histological body donors was 78%.

Conclusions

Our findings support the presence of SPS in the majority of body donors. To our knowledge, this is the first histological study of the SPS.

## Introduction

The dural venous sinuses are the termination sites into which the veins of the brain drain. The sphenoparietal sinus (SPS), also referred to as the lesser sphenoid wing sinus, the superior sphenoidal sinus, the sinus alae parva, and the sinus of Breschet, collects venous blood from the anterior temporal lobe and receives tributaries from vessels including the superficial Sylvian, middle meningeal, and anterior temporal diploic veins to then drain into the cavernous sinus [1-3].

Publications from the 19th century to the present contain conflicting opinions regarding the SPS - some drawing contradictory conclusions on the existence of the structure entirely. The controversy surrounding the SPS is likely due to the relative paucity of research on its anatomy. For instance, Knott [4] mentioned that he was "surprised that a description [of the SPS] is so often omitted from our textbooks". Given the inconsistent descriptions and relative lack of anatomical investigations, the present anatomical study was performed to better elucidate this structure of the skull base.

## Materials and methods

Ten embalmed and latex-injected adult body donors' heads (20 separate sides) underwent microdissection of the SPS using a surgical microscope (Zeiss, Germany). The body donors had given their written informed consent for their use for scientific purposes prior to death. According to National Law, scientific institutions (in general institutes, departments, or divisions of medical universities) are entitled to receive the body after death mainly by means of a specific legacy, which is a special form of last will and testament. No bequests are accepted without the donor having registered their legacy and been given appropriate information upon which to make a decision based upon written informed consent (policy of ethics) [5]. As the tissues used are cadaveric, our institution does not require an Institutional Review Board approval and is, thus, exempt.

The average age at death of the body donors was 77.5 years (range 54 to 91 years). Eleven body donors were male, and nine were female. The left and right internal jugular veins were cannulated with rubber tubing, and blue-colored latex was injected into both veins until resistance was met. The specimens were allowed to cure in a cooler for one week. The lesser wing of the sphenoid was removed with bone rongeurs. The gross observations of the SPS, including presence and size, were documented. Measurements of the SPS were made with microcalipers (Mitutoyo, Japan). All measurements were made three times, and the average was taken. In ten additional body donors (20 sides), the entire dura on the underside of the lesser wing of the sphenoid bone that covers the region known as the groove for the SPS was harvested from each body donor and submitted for histological analysis - hematoxylin&eosin (H&E), Periodic acid-Schiff​​​​​​ (​PAS), Mason Trichrome). For controls, five left and five right transverse sinuses were harvested and analyzed histologically. Statistical analysis was performed with a significance set at p<0.05. The authors state that every effort was made to adhere to all local and international ethical guidelines and laws that pertain to the use of human body donors in anatomical research [6].

## Results

**Figure 1:**
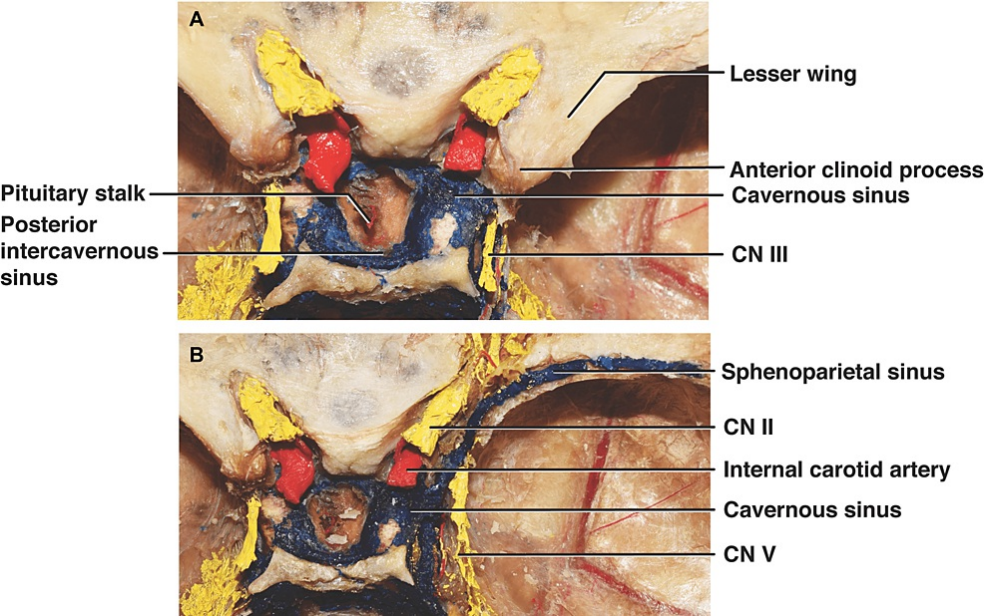
Step-by-step cadaveric dissection of the right sphenoparietal sinus Before (A) and after (B) the lesser wing of the sphenoid was removed. CN - cranial nerve

A definitive SPS was identified in 14 (seven left and seven right sides; 70%) of the latex-injected body donors (Figure 1).

**Figure 2:**
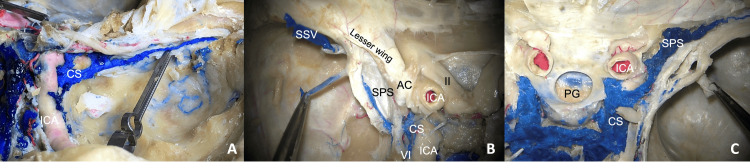
Three body donors noting the sphenoparietal sinus The left image (A) shows a medium-sized left-sided sinus draining into the cavernous sinus (CS). The middle image (B) shows a small left-sided sinus with the superficial Sylvian vein (SSV) and anterior temporal vein (forceps) draining into it. The right image (C) shows a large right-sided sinus (SPS) with a relatively large anterior temporal vein (forceps) draining into it. Note the internal carotid artery (ICA), pituitary gland (PG), abducens nerve (VI), optic nerve (II), and anterior clinoid process (AC).

When present, the sinuses were classified as small (less than or equal to 3 mm diameter; 13 sides), medium (less than or equal to 5 mm diameter; six sides), or large (greater than 6 mm in diameter; three sides). Tributaries included the middle meningeal veins, superficial Sylvian vein, and anterior temporal veins (Figure 2).

**Figure 3:**
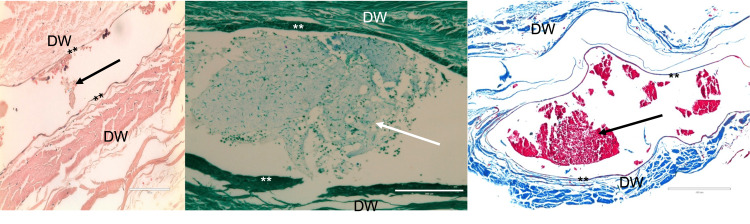
Histological examples through the sphenoparietal sinus (A) H&E x 400, (B) PAS x 200, (C) Masson trichrome x 100. Note the blood cells in the lumen of each body donor (arrows). **endothelial lining; DW - dural wall

**Figure 4:**
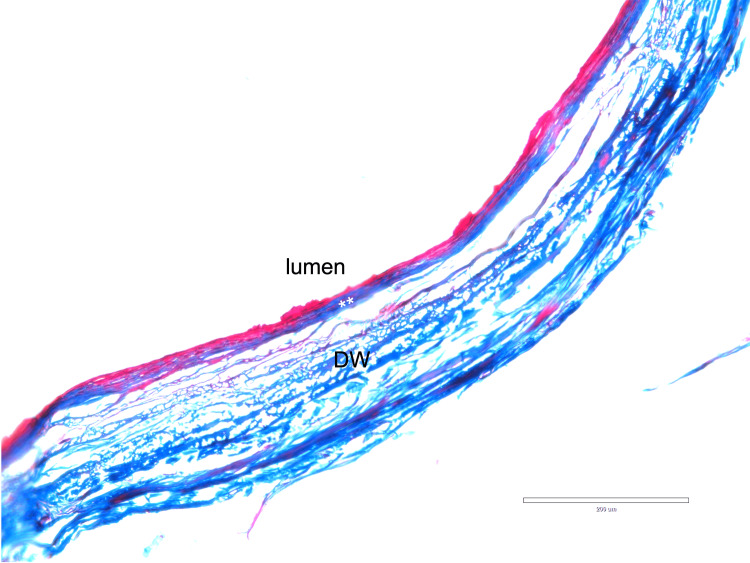
Histological example of the wall of the left transverse sinus (Masson trichrome x 20) ** endothelial lining; DW - dural wall

All sinuses drained medially into the cavernous sinus either above or below the ophthalmic nerve. For the body donors analyzed histologically, 17 (85%; nine left sides and eight right sides) were consistent with a dural venous sinus and not a vein and were observed to have a rich nerve and arterial supply within their walls (Figure 3). There were no major histological differences noted between the SPS and transverse sinuses (Figure 4). All sinuses were found on histology to have an external wall of the dura mater lined by endothelium.

The combined prevalence of the sinus via gross and histological body donors was 78%. None of the body donors was found to have gross intracranial pathology or major anatomical variations at the skull base. Statistically, no differences were found comparing the SPS on the left and right sides or between male and female body donors.

## Discussion

**Figure 5:**
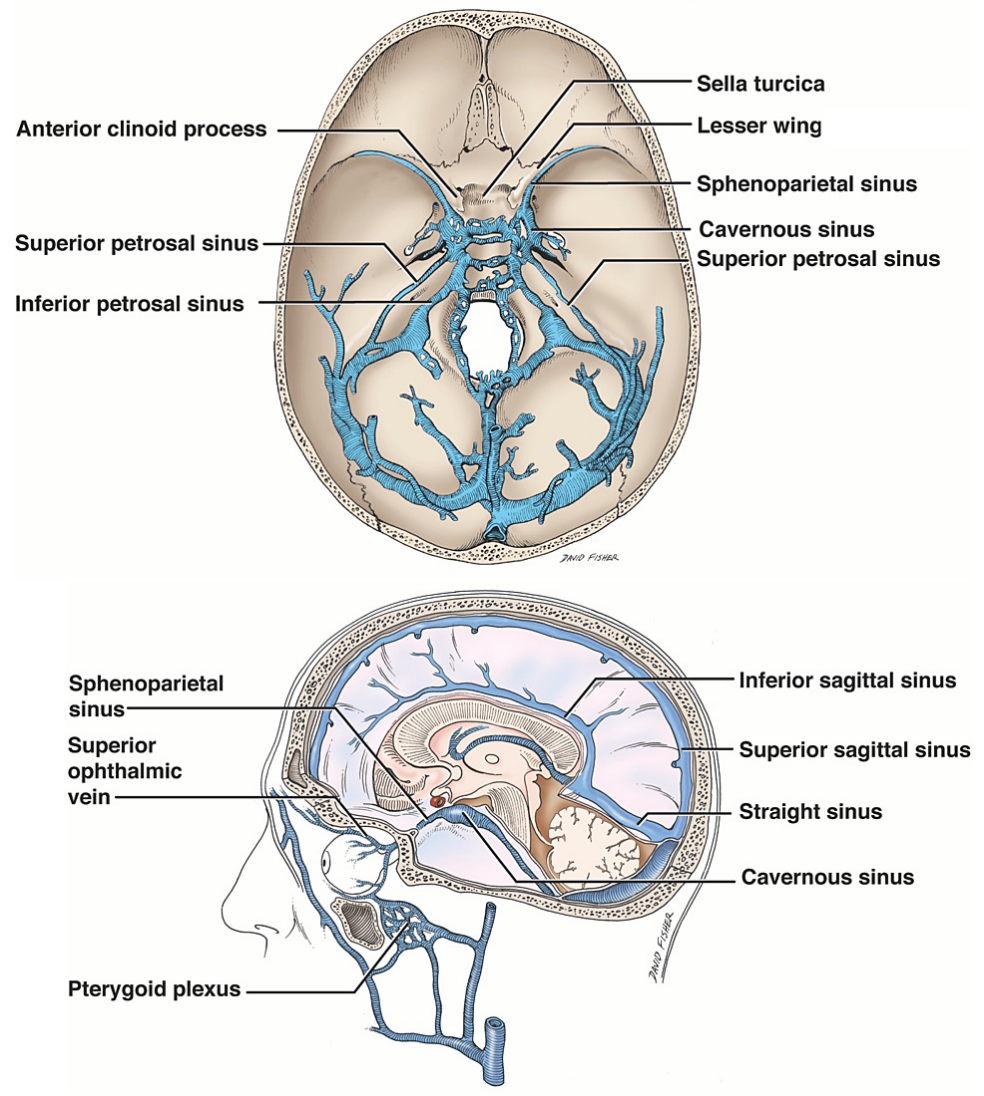
Schematic drawings of the dural venous sinuses of the skull base noting the position of the sphenoparietal sinus Composed by authors.

Via both microdissection and histological analysis, we identified SPS in the majority of body donor sides; however, the size of the sinus was variable. This, combined with its occasional absence, likely contributes to its frequent omission from discussion of the skull base dural venous sinuses (Figure 5) as well as controversies regarding its existence.

Embryology

The SPS is derived from the anteroparietal meningeal sinus, which consists of the middle meningeal vein and part of the embryonic pro-otic sinus [7]. The SPS is formed by the merging of the anteroparietal meningeal sinus and the cranial remnant of the tentorial sinus. The tentorial sinus regresses as the cerebral hemispheres grow and may become part of the cavernous sinus. Alternatively, its cranial portion may persist as a paracavernous sinus that forms the SPS. Furthermore, the ventral end of the SPS may be continuous with the temporal diploic veins from which it is derived, subsequently merging with the ophthalmo-meningeal sinus [7].

The SPS has been described as resembling venae comitantes due to the fact that the middle meningeal veins drain the lateral dura and travel closely between branches of the middle meningeal artery and the bone [8]. The formation of the SPS is partly diploic as a result of the anastomosis of diploic veins and scalp veins in the anterior temporal region. The formation may also be considered meningeal due to the anastomosis of anterior meningeal veins with the superficial Sylvian vein, which eventually drains into the cavernous sinus [7,9]. It is also worth noting that the SPS is unique to hominoids and evolutionarily only observed in Homo and Pongo [10].

Pathology

Dural arteriovenous fistulas (dAVFs) involving the SPS are a rare form of intracranial vascular malformation. Most reported fistula cases involve the transverse, sigmoid, or cavernous sinuses [11]. The first documented case of a dAVF involving the SPS was described by Pakarinen [12] in a 25-year-old male who suffered head trauma, subsequently presenting with a headache and bruit on the ipsilateral side of the dAVF involving the middle meningeal artery and SPS. Other cases of dAVFs involving the SPS after head trauma have also been reported [12-14]. For instance, a 37-year-old patient with increased intracranial pressure post-head trauma was found to have a dAVF between the middle meningeal artery and the SPS [13]. Due to the irreversible damage, the patient was not treated surgically and died six days later. Another case of a fistula between the middle meningeal artery and SPS after head trauma was reported in a 53-year-old man who had suffered multiple brain contusions and a temporoparietal bone fracture [14]. This patient was diagnosed with increased intraocular pressure and developed symptoms of cavernous sinus syndrome that immediately resolved following the endovascular coil embolization of the fistula. Bitoh et al. [15] reported a case of a 14-year-old male with a dAVF between the SPS and meningeal branches of the left external carotid artery.

Numerous cases of spontaneous dAVFs have also been reported. One such fistula was identified in a 54-year-old man who was found to have a fistula between the middle meningeal artery and SPS [11]. As previously mentioned in a case by Macdonald et al. [16], diagnosis of SPS dAVF was delayed due to the non-specific nature of the patient's symptoms, i.e., facial swelling and tenderness - all of which were attributed to more common pathologies. Although SPS fistulas can have myriad clinical manifestations, they are not associated with subarachnoid hemorrhage or intracranial venous hypertension [11].

Lastly, cerebral venous thrombosis (CVT) is a rare cause of stroke associated with just 0.5% of reported cases that most commonly occurs within the dural venous sinuses, specifically the superior sagittal and the transverse sinuses [17,18]. At least two cases of CVT involving the SPS have been reported. Di Caprera et al. [18] described a case of a 38-year-old woman who presented with an intractable headache of one-week duration. MRI venogram revealed venous thrombosis of the left SPS. Interestingly, the other reported case of CVT occurred in the left SPS of a 32-year-old woman who presented with headache, new-onset complex-partial seizure, and emesis [17]. Both aforementioned patients were successfully treated with anticoagulant therapy.

Interventional procedures

The use of a wedge microcatheter for transarterial embolization using liquid embolic agents has proved beneficial in the treatment of complex dAVFs in which there direct cortical venous drainage or transvenous embolization cannot be performed [11,19]. In a study conducted by Nelson et al. [19], 21 patients were treated with the liquid embolic agent, transarterial N-butyl cyanoacrylate (NBCA), using a wedged microcatheter. One of the patients presented seizures and was found to have a dAVF involving the SPS. After NBCA treatment, occlusion of the dAVF was confirmed via follow-up angiography, and the neurological exam was intact 16 weeks postoperatively. Khadavi et al. [11] also described a case of a 54-year-old man who presented with sudden-onset glaucoma and proptosis. The evaluation identified a dAVF between the middle meningeal artery and a sphenoidal venous branch which drained secondarily into the cavernous venous sinus. A microcatheter was used to catheterize the left middle meningeal artery at the tip of the arteriovenous (AV) fistula, and ethylene vinyl alcohol (EVOH) copolymer, onyx was injected into the proximal draining vein to occlude the AV communication. Immediately after the procedure, the patient noted the resolution of the symptoms. Additionally, Macdonald et al. [16] reported a case of dAVF hemorrhage involving the SPS in a 42-year-old man who was treated with transarterial embolization. At the six-month follow-up, the patient denied any symptoms, and repeated angiography confirmed occlusion of the fistula.

Skull base surgery and imaging

Anatomical knowledge of the SPS is important during various approaches to the skull base. Understanding the SPS enables surgeons to choose an appropriate procedural approach in the treatment of skull base pathologies and to avoid complications, including brain edema secondary to damage of the SPS if it is not carefully dissected from its attachment to the lesser wing of the sphenoid [2,15]. Anatomical awareness of the SPS may also assist when performing procedures such as the so-called SPS transposition, as described by Niibo et al. [20], which allows for the displacement of bridging veins from the temporal pole. Approaches to the lateral aspect of the cavernous sinus for pathology extending into the superior orbital fissure will both benefit from insight regarding the anatomic relationships of SPS.

Fat-suppressed contrast-enhanced 3D fast gradient echo-MR imaging has proven to be effective and superior to traditional CTA methods. It provides better images of vessels adjacent to or penetrating bony structures, which are difficult to obtain with 3D CTA. Thus, contrast-enhanced fat-suppressed 3D imaging allows a clear depiction of venous structures, including the SPS, in healthy subjects as well as those with dAVFs [1,21].

Declaration

The authors sincerely thank those who donated their bodies to science so that anatomical research could be performed. These donors and their families deserve our highest gratitude [22].

## Conclusions

Our findings support the definitive presence of SPS in the majority of body donors. However, there is variability in the size of such venous structures. Although the sinus can be absent, it can also, as occurred in our study, frequently be of a small caliber. Taken together, these data illustrate why some have concluded that the sinus might not exist. For the skull base surgeon, the sinus should be expected in the majority of patients but will often be of diminutive size unless influenced by pathology. To our knowledge, this is the first histological study of the SPS.
